# Retinal pigment epithelium stress following intravitreal ganciclovir: a novel insight from clinical spectrum

**DOI:** 10.1186/s12348-025-00532-3

**Published:** 2025-09-26

**Authors:** Kui Fang Du, Xue Hui Shi, Chang Ping Zhang, Hong Wei Dong, Wen Jun Kong, Jost B. Jonas, Wen Bin Wei, Ya Xing Wang

**Affiliations:** 1 Department of Ophthalmology, Beijing Youan Hospital, Capital Medical University, Beijing, China; 2 Beijing Ophthalmology and Visual Science Key Lab, Beijing Key Laboratory of Intraocular Tumor Diagnosis and Treatment, Beijing Tongren Eye Center, Beijing Tongren Hospital, Capital Medical University, Beijing, China; 3 Department of Pharmacy, Beijing Youan Hospital, Capital Medical University, Beijing, China; 4 Department of Ophthalmology, Medical Faculty Mannheim of the Ruprecht-Karis-University, Mannheim, Germany; 5 Beijing Visual Science and Translational Eye Research Institute (BERI), Beijing Tsinghua Changgung Hospital, Tsinghua Medicine, Tsinghua University, 168 Litang Road, Changping District, Beijing, 102218 China

**Keywords:** Ganciclovir, Intravitreal injection, Cytomegalovirus retinitis, Retinal pigment epithelium

## Abstract

**Objective:**

The retinal toxicity of intravitreal ganciclovir (GCV) is contentious. Our study aims to describe new clinical findings following intravitreal GCV and propose a mechanism for the pathogenesis of those post-injection retinal changes.

**Methods:**

A retrospective case series included 114 patients with cytomegalovirus retinitis (CMVR) receiving intravitreal GCV (2.5 mg/0.05 mL) combined with systemic therapy. Patients with presumed retinal toxicity following intravitreal GCV injection were enrolled. Clinical data, laboratory tests, ocular examination, and multimodal images of the retina were collected. Serial optical coherence tomography (OCT) was performed to monitor pre- and post-retinal changes.

**Results:**

Five patients (7 eyes) (4.4%±2.6%) with macular spared from CMVR lesions experienced acute vision loss and transient macular edema after the GCV injection; 1 patient (0.9%±2.6%) with bilateral CMVR developed chronic and progressive retinal pigment epithelium (RPE) atrophy in the CMVR-spared areas of the eye receiving repeated GCV injections, contrasting with untreated contralateral eyes. Three of them were additionally diagnosed as cytomegalovirus-immune recovery retinitis. With OCT, all eyes showed intact Bruch’s membrane and normal thickness of inner retinal layers. Seven eyes with acute vision loss showed outer retinal changes within 24 h post-injection, including macular SRF (7 eyes), macular vertical hyperreflective foci (5 eyes), and ONL edema (4 eyes), and resolving spontaneously weeks later.

**Conclusions:**

Intravitreal GCV treatment may trigger acute macular edema and chronic RPE degeneration in CMVR patients. We hypothesize that RPE dysfunction is the pivotal pathogenic mechanism, potentially induced by multifactorial interplay, including chemical irritation, cumulative exposure, systemic comorbidities, and ocular inflammatory cascades.

**Supplementary Information:**

The online version contains supplementary material available at 10.1186/s12348-025-00532-3.

## Introduction

The intravitreal route has become a cornerstone in the management of sight-threatening ocular diseases. Ganciclovir (GCV), a potent antiviral agent, is widely employed via intravitreal injection to treat viral retinitis, including cytomegalovirus retinitis (CMVR) and acute retinal necrosis while minimizing systemic toxicity [1, 2]. Globally, CMVR remains a cause of blindness among people living with human immunodeficiency virus (HIV) and HIV-negative patients [3, 4], such as advanced age, diabetes mellitus, severe immune dysfunction, and congenitally infected infants. In these settings, intravitreal GCV injections are often the most critical accessible therapy due to the limited availability of alternatives and rely on frequent injections (weekly to biweekly) and prolonged treatment courses.

In recent years, novel GCV prodrugs [5] or innovative drug delivery systems—such as transferrin conjugated liposomes [6] and nanoparticles [7]—have emerged as promising strategies to prolong intraocular drug half-life and enable site-specific delivery to retinal lesions and theoretically mitigate procedure-related complications like vitreous hemorrhage or iatrogenic cataract. Nevertheless, debates persist regarding the retinal damage profile of intravitreal GCV. Sporadic case studies describe macular edema and retinal pigment epithelium (RPE) atrophy following intravitreal GCV injection [8, 9], while animal studies suggest transitory retinal edema [10]. Despite growing enthusiasm for novel GCV formulations, translational research remains disproportionately focused on optimizing antiviral efficacy and pharmacokinetics, often overlooking comprehensive clinical safety evaluations. This gap underscores the urgent need for real-world evidence to delineate the spectrum of GCV-associated retinal complications and inform risk-benefit paradigms.

Herein, we present a retrospective analysis of consecutive six cases demonstrating presumed retinal toxicity following intravitreal GCV. We hope to explore possible pathogenesis of GCV-associated retinal complications, and underscore a cautionary note for clinical strategies and researches of novel GCV formulations.

## Methods

This study was a retrospective observational case series with presumed GCV toxicity at the Ophthalmology Department of Beijing Youan Hospital between January 2017 and December 2023.

The presumed retinal toxicity following intravitreal GCV injection was defined as retinal structural alterations occurring post-injection that could not be attributed to other etiologies (e.g., procedure-related complications, CMVR disease progression, or other systemic comorbidities). Inclusion Criteria: Diagnosis of CMVR depended on medical history, laboratory assessment (e.g., positive CMV DNA in aqueous humor), and fundus examination; treatment with intravitreal GCV injection; availability of spectral-domain optical coherence tomography (OCT) imaging before and after injection; follow-up period of at least 3 months post-injection. Exclusion Criteria: Patients without post-injection OCT records or sufficient follow-up data.

We collected medical records from all patients, including demographic data, systemic comorbidities, laboratory tests, medication use, ophthalmic examination (pre- and post-injection), and treatment. For intravitreal GCV injection, we collected records about the GCV dose, the injection frequency, and the cumulative injections. For ophthalmic examinations, we collected records and ophthalmic imaging data before and after each intravitreal GCV injection, including best-corrected visual acuity (BCVA), intraocular pressure, slit-lamp microscopy, fundus photography (OPTOS^®^ Daytona), and spectral-domain OCT (RS-3000, Nidek, Gamagori, Japan).

We conducted statistical analyses using IBM SPSS version 29.0. We tested the proportions of patients with presumed retinal toxicity using summary statistics, including proportions and standard errors. Furthermore, we used different indicator strips to test the pH value of the intravitreal GCV solution used in these cases.

## Results

Among 114 patients included in this study, five patients (7 eyes) (4.4%±2.6%) experienced acute vision loss and macular changes after GCV injection; 1 patient (1 eye) (0.9%±2.6%) experienced chronic RPE degeneration after GCV injection. The demographic data of these six patients (four male and two female, age range 28–73 years, 7 eyes) and clinical presentations are summarised in Tables 1 and 2. All patients were diagnosed with acquired immune deficiency syndrome (AIDS) and CMVR. They were treated with conventional intravenous GCV (5 mg per kilogram, every 12 h) combined with intravitreal ganciclovir injection (2.5 mg/0.05 mL), one or two injections every week. Three patients belong to cytomegalovirus-immune recovery retinitis (CMV-IRR) in consideration of antiretroviral treatment (ART) for 4–12 months and CD4^+^T cell recovery (≥ 100/µL) before CMVR deterioration.

**Table 1:** Summary of clinical characteristics of all cases in this study

Case	Gender	Diagnosis	Incident eye	Contralateral eye	Initial BCVA	Onset time	BCVA after incident injection	IOP (mmHg)	After which injection did the symptom appear?	Management	Was there any worsening after subsequent injection(s)?	Follow-up (months)
1	Male	CMVR; AIDS; DLBCL; hypoproteinemia; anemia	OD	**	1.0	POD 1 after patch removal	0.25	7	6th	†	no	12
2	Male	CMV-IRR; AIDS; hypoproteinemia	OD	-	0.8	Postoperative 2 h after patch removal	0.3	17	1st	†	no	36
			OS		0.6	Postoperative 2 h after patch removal	0.2	11	1st	†	no	36
3	Male	CMV-IRR; AIDS; pulmonary tuberculosis; tubercular meningitis; hypoproteinemia; anemia	OD	-	0.6	POD 1 after patch removal	0.2	12	3rd	†††	N/A	9
			OS		0.6	POD 1 after patch removal	0.3	15	3rd	††	N/A	9
4	Female	CMVR; AIDS; PCP; hypoproteinemia	OS	*	0.4	POD 1 after patch removal	0.02	12	5th	††	N/A	27
5	Male	CMVR; AIDS; hypoproteinemia; anemia	OD	***	0.6	POD 1 after patch removal	0.2	10	4th	††	N/A	9
6	Male	CMV-IRR; AIDS; hyperlipemia	OD	***	0.15	4 weeks after the final injection	N/A	12	6th	††	N/A	3

AIDS: acquired immune deficiency syndrome; CMVR: ytomegalovirus retinitis; CMV-IRR: cytomegalovirus-immune recovery retinitis; DLBCL: diffuse large B cell lymphoma; N/A: not available; PCP: pneumocystis carinii pneumonia; POD: Postoperative Day; RD: retinal detachment

* Normal

** Eyes with CMVR, and intravitreal ganciclovir injection

*** Eyes with CMVR, but no intravitreal ganciclovir injection

† The conventional intravitreal ganciclovir injection was continued, and no further treatment was applied

†† The conventional intravitreal ganciclovir injection was stopped, but no further treatment was applied

††† The conventional intravitreal ganciclovir injection was discontinued, and an intravitreal injection of anti-vascular endothelial growth factor (anti-VEGF) agents was administered

**Table 2:** Summary of retinal presentations and OCT findings when patients had acute vision loss after intravitreal ganciclovir injection

Case	Lowest CD4 + T (/µL)	Latest CD4 + T (/µL)	aqueous CMV-DNA (copies/µL)	Duration of ART (months)	Involved eye	Visible vitreous opacity	Cotton wool spot	OCT findings							Figures
								Macular PVD	Vitreous cells	Macular SRF	Macular hyperreflective foci	Other changes	Resolution of macular changes	Duration of macular changes (weeks)	
1	13	58	1560	10	OD	No	No	No	No	Yes	No	RPE fold	Yes	2	Supplemental figure 1
2	0	200	254,000	12	OD	Yes	No	No	No	Yes	Yes	ONL edema	Yes	5	Figure 1
			112,500		OS	Yes	No	No	No	Yes	Yes	ONL edema	Yes	5	Supplemental figure 2
3	48	224	15,040	8	OD	No	No	No	No	Yes	Yes	ONL edema	Yes	6	Supplemental figure 3
			6827		OS	No	No	No	No	Yes	No	No	Yes	1	Supplemental figure 4
4	3	13	822,000	0	OS	Yes	No	No	Yes	Yes	Yes	No	Yes	3	Supplemental figure 5
5	21	21	8320	0	OD	Yes	Yes	No	Yes	Yes	Yes	ONL edema	Yes	20	Figure 2
6	19	130	26,000	4	OD	Yes	N/A	N/A	N/A	N/A	N/A	RPE atrophy; granular deposits	N/A	N/A	Figure 3

*ART* Antiretroviral treatment, *CMV* Cytomegalovirus, *DNA* Deoxyribonucleic acid, *N/A* Not available, *OCT* Optical coherence tomography, *OD* Oculus dexter, *ONL* Outer nuclear layer, *OS* Oculus sinister, *PVD* Posterior vitreous detachment, *SRF* Subretinal fluid

**Fig. 1:**
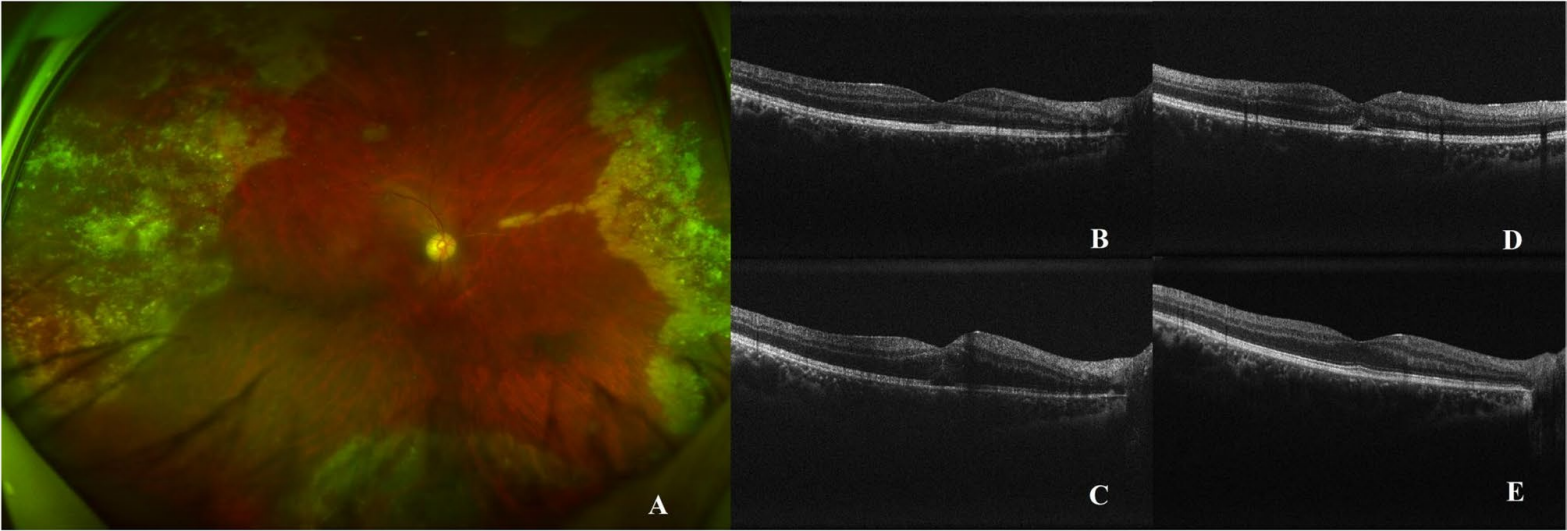
CMVR without macular involvement in the right eye of Case 2 (**A**). OCT image before the incident injection (**B**). Two hours after the first intravitreal ganciclovir injection (2.5 mg/0.05 mL), the patient reported impaired vision, with OCT showing outer nuclear layer (ONL) edema and macular subretinal fluid (**C**). On postoperative day 3, the ONL edema resolved, but macular subretinal fluid remained (**D**). At 5 weeks post-injection, macular subretinal fluid resolved, and OCT demonstrated restored macular architecture (**E**)

**Fig. 2:**
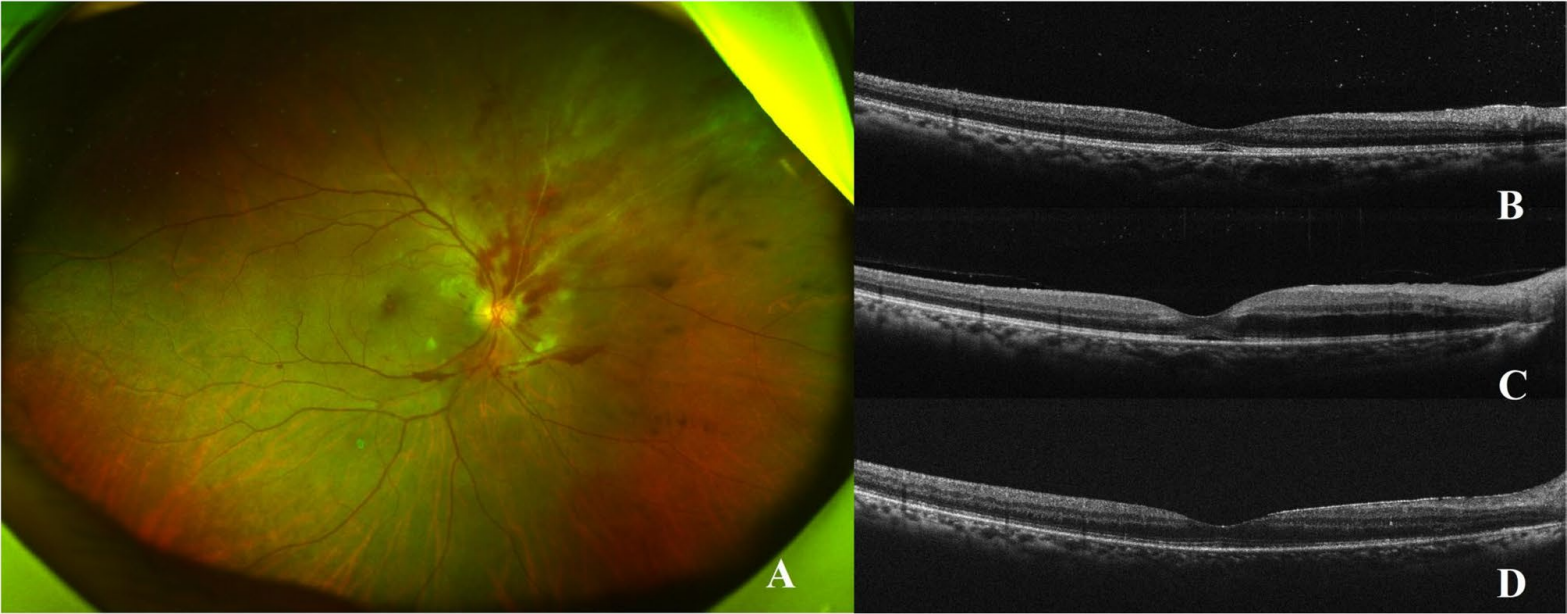
CMVR without macular involvement in the right eye of Case 5 (**A**). OCT image before the incident injection (**B**). On the first day after the 5th intravitreal ganciclovir injection (twice weekly, 2.5 mg/0.05 mL), the patient experienced impaired vision, with OCT revealing macular subretinal fluid and outer nuclear layer edema (**C**). Subsequent injections were discontinued. At 20 weeks post-injection, OCT showed restored macular architecture (**D**)

**Fig. 3:**
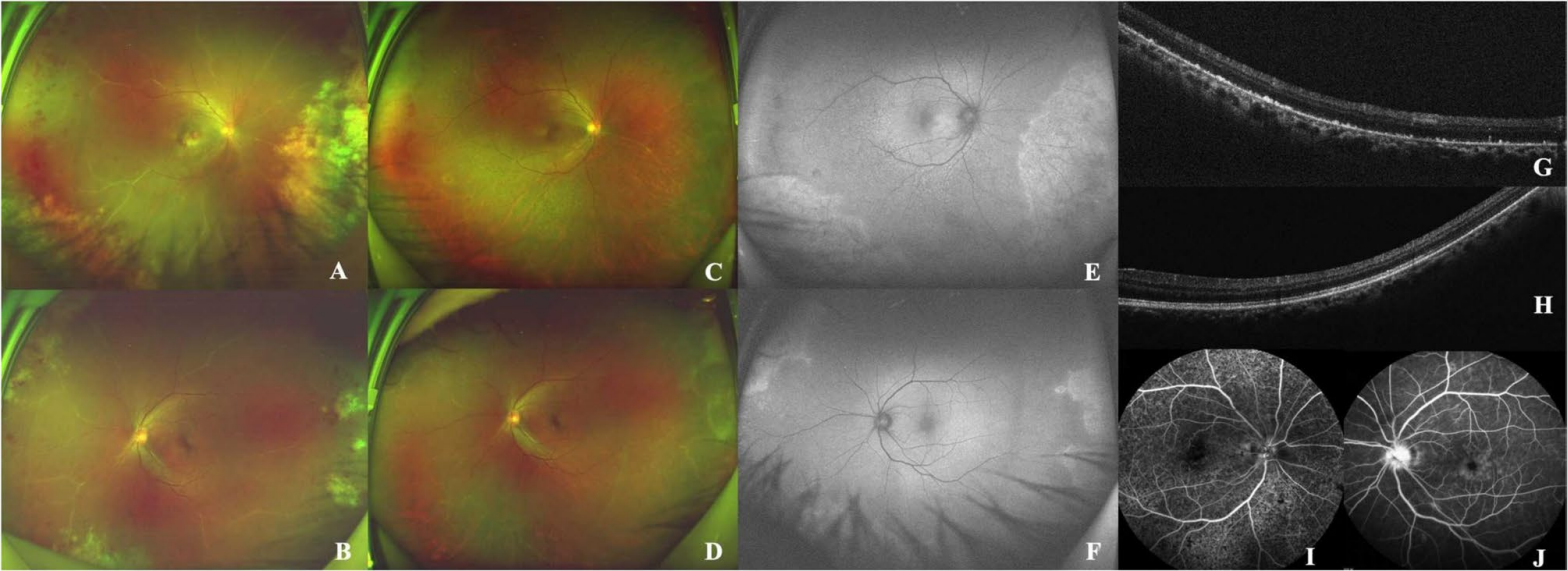
CMVR with macular involvement in the right eye of Case 6 (**A**); CMVR without macular involvement in the left eye of Case 6 (**B**). The right eye received systemic ganciclovir infusion combined with intravitreal ganciclovir injections (twice weekly, 2.5 mg/0.05 mL, a total of six injections). At 3 months post-treatment, the patient experienced vision improvement in both eyes, but the right eye exhibited mottled pigmentary changes in the mid-peripheral retina unaffected by CMVR (**C**: fundus image, **E**: autofluorescence); the left eye showed resolved CMVR lesions without pigmentary changes (**D**: fundus photo, **F**: autofluorescence). OCT of the right mid-peripheral non-CMVR area revealed RPE depigmentation and punctate hyperreflective foci (**G**), while the left eye showed no abnormalities (**H**). FFA of the right eye demonstrated speckled RPE defects (**I**), whereas the left eye showed no pigmentary abnormalities (**J**)

Seven eyes presented acute vision loss on the first time patch removal. These acute vision losses occurred after a single session within a series of repeated intravitreal injections. The complete set of fundus images and progression in OCT, from case 1 to case 5, were presented in Figs. 1, 2 and 3 and online **s**upplemental Figs. 1–5. Seven eyes showed acute macular changes in OCT: 7 eyes with macular SRF, 5 eyes with macular vertical hyperreflective foci, and 4 eyes with ONL edema. All these acute macular changes showed intact Bruch’s membrane and normal thickness of inner retinal layers and were resolved after close monitoring for weeks (range 1–20 weeks). None of them showed vitreomacular traction in OCT.

The sixth patient with CMV-IRR revealed bilateral anterior chamber inflammation, mutton-fat keratic precipitates, and moderate vitreous haze with CMVR lesions. One month after six consecutive intravitreal GCV injections (administered twice weekly) in the right eye, CMVR lesions resolved; however, mottled RPE alterations emerged in retinal areas previously unaffected by CMVR. OCT demonstrated RPE atrophy and granular hyperreflective deposits in all quadrants. Fundus autofluorescence (AF) exhibited stippled hyper-AF and hypo-AF, while fundus fluorescein angiography (FFA) showed stippled hyperfluorescence and hypofluorescence. Notably, the contralateral eye, which also had CMV-IRR, did not receive intravitreal GCV and exhibited no RPE changes. Over a subsequent 4-month follow-up, while the macular CMVR lesions resolved entirely with improved visual acuity, the RPE changes progressively worsened.

Notably, these pH indicator strips revealed a strongly alkaline solution of the GCV solution (2.5 mg/mL) with a pH of 10–11 (Supplemental Fig. 6).

## Discussion

In these AIDS-related CMVR cases with intravitreal GCV injection, we observed paradoxical retinal changes, including acute macular edema and chronic RPE degeneration. The earliest OCT changes can be observed as early as 2 h postoperatively. These incidental abnormalities in non-lesional areas lead to patient-reported vision impairment, potentially triggering distrust and exacerbating tensions in clinical interactions.

Two critical questions arise: Are they related to intravitreal GCV? Secondly, what are the possible mechanisms for these acute macular edema and chronic RPE degeneration?

### Clinical correlates: temporal association with ganciclovir

Our observation of acute macular edema in 4.4% of treated eyes and chronic RPE alterations in 0.9% underscores the need to reevaluate the safety profile of this widely used therapy. The temporal association between intravitreal GCV injections and acute macular changes or chronic RPE damages suggests drug induction rather than CMVR progression. Table 3 summarizes the previous incidental cases with presumed retinal toxicity after intravitreal GCV injection with therapeutic dose, including macular infarction, acute macular edema, crystal formation in the vitreous, and chronic RPE atrophy [8, 9, 11–15]. 

**Table 3:** Clinical characteristic of previous patients with persumed retinal toxicity after intravitreal ganciclovir injection with therapeutic dose

Case	Reference	Gender	Diagnosis	Systemic anti-CMV treatment	Intravitreal GCV dose	Symptoms	Anterior chambers signs	Fundus signs	OCT signs	Other signs	Management	Prognosis
1	An et al.(2024) [8]	Female	CMVR; localized RD; acute lymphocytic leukemia	Oral VGCV	2.0 mg/0.1 mL (1 injection)	None	VA: 20/40	RPE depigmentation 1 month post-injection	Outer retinal layer thinning with multiple hyperreflective foci in the RPE	Speckled RPE defects in FFA; flat amplitudes in full-field ERG; severely depressed VF	Several IVTAs	Progressive enlargement and clumping of RPE atrophic areas
2	Liu et al. (2022) [9]	Male	CMVR; DLBCL	No	4 mg	Vision loss 2 h after the 4th injection	VA: 20/1000	Nothing unusual except CMVR lesions	CME; SRF; hyperreflective foci	Fuorescence leak in the macula and paramacular in FFA	Close monitoring	VA improved to 20/400; No macular abnormalities in OCT
3	Kim et al. (2008) [11]	Male	ARN	Intravenous acyclovir and foscarnet	2 mg/0.05 mL	Vision loss One day after the 2nd injection	VA: NLP;	Severe optic disc swelling and macular infarction	N/A	FFA: normal arm-to-retina and arteriovenous transit time; macular infarction	N/A	No VA improvement
4	Iu et al. (2018) [12]	Male	CMVR; bullous pemphigoid (long term oral steroid)	Oral VGCV	2.5 mg/0.05 mL	No ocular pain or visual change	VA: 20/60; IOP: 11 mmHg; mild anterior chamber cells	Intraoperative needle-shaped golden-yellow crystals in vitreous	N/A	Same condition, only one eye showed the issue.	Close monitoring	The crystals dissolved spontaneously after 5 min.
5	Choopong et al. (2010) [13]	Female	CMVR; chronic anterior uveitis	No	4 mg/0.04 mL	Immediate vision loss after the 1 st injection	VA: FC at 1 foot; IOP: 33 mmHg;	Crystal formation in the vitreous; retinal edema; cherry red spot in the macula	N/A	CRVO and CME in FFA	Vitrectomy; intravitreal TA injection	Optic nerve atro phy without VA improvement
6	Kapoor et al. (2024) [14]	Male	CMVR; Granulomatosis	Oral VGCV was stopped	2.5 mg/0.05 mL (27 injections)	Slowly vision loss	VA: 20/200	(3 months later) progressive RPE atrophy in all quadrants; healed CMVR lesions	Retinal atrophy in the entire retina; loss of photoreceptors	Extinguished scotopic and photopic in ERG	N/A	Progressive RPE atrophy
7	Yeh et al. (2010) [15]	Female	CMVR; AIDS	No	intravitreal GCV implantation and foscarnet injections	N/A	VA: 20/200	(2 weeks later) RPE depigmentation; healed CMVR lesions	N/A	Stippled hyper-AF and hypo-AF in the entire retina in FAF	N/A	Progressive RPE changes

*AF* Autofluorescent, *CMVR* Cytomegalovirus retinitis, *CME* Cystoid macular edema, *CRVO* Central retinal artery occlusion, *DLBCL* Diffuse large B cell lymphoma, *ERG* Electroretinogram, *FAF* Fundus autofluorescent, *FC* Finger count, *FFA* Fluorescent fundus angiography, *GCV* Ganciclovir, *HM* Hand movements, *LP* Light perception, *N/A* Not available, *NLP* No light perception, *IVTA* Intravitreal triamcinolone, *RD* Retinal detachments, *RPE* Retinal pigment epithelium, *SRF* Subretinal fluid, *IOP* Intraocular pressure, *VA* Visual acuity, *VF* Visual field, *VGCV* Valganciclovir

Based on the temporal profiles of acute macular edema in our case series, we postulate that the onset of macular edema likely occurs immediately following the injection. Partial resolution of edema was observed upon removal of the ocular patch on postoperative day (POD) 1, followed by gradual spontaneous resolution in subsequent days. These acute macular changes and temporal profiles totally align with previous case reports with overdose or normal doses of intravitreal GCV [9, 16]. The temporal concordance of OCT patterns between delayed patch removal (POD 1) and early removal cohorts (2 h post-operation) implies that the transient macular edema might be initiated during the immediate postoperative phase.

The delayed-onset RPE mottling and atrophy are observed in Case 6 and persist despite CMVR resolution. These RPE degenerations aligns with previouse case reports and were taken as retinal toxicity from intravitreal GCV [8, 14, 15]. This hypothesis is strengthened by the absence of such changes in the untreated contralateral eye from previouse cases, as well as our report.

### Mechanistic insights: RPE dysfunction

OCT imagings of these cases reveal macular SRF, ONL edema, and macular hyperreflective foci, with Bruch’s membrane integrity and normal thickness of inner retinal layers. We propose RPE cells as the pivotal pathogenic hub orchestrating disease progression through their central role in barrier function, transportation of nutrients, ions, and water [17]. The observed chronic and diffuse RPE atrophy also provides pathophysiological substantiation to this hypothesis.

Extracellular fluid accumulates in the cavity between the neural retina and the RPE layer, forming the SRF in each case, accompanied by the increase of intracellular fluid volume (cell swelling) in ONL in some cases. The macular ONL is formed by nuclei, inner and outer segments of cone photoreceptors. Since the macular fovea is avascular, the choroidal vessels supply nutrients and oxygen to the high energy-demanding ONL [18]. RPE cells are vital barriers for substrate exchange between choroid and ONL: selectively transport fatty acids, ascorbic acid, glucose, and other nutrients from choroids to photoreceptors; transport metabolic end products, ions, and excess water from photoreceptor cells to the choroid [17]. Intracellular edema in ONL is thought to be a consequence of metabolic disturbances. Thus, the RPE dysfunction could be the most plausible explanation for both macular SRF and ONL edema.

### Multifactorial contributors to RPE dysfunction

#### Ganciclovir contributor

The retinal toxicity of GCV is contentious. Previous animal studies on intravitreal GCV injections demonstrated dose-dependent retinal toxicity in rabbit eyes. Following administration of different doses (200–600 µg/0.1mL), electroretinography responses were completely abolished at 600 µg or significantly impaired at lower doses, which persisted for up to 4 months. Electron microscopy showed degenerative changes across all retinal layers, particularly prominent in the ONL [10]. At a higher dose of GCV (2 mg/0.1mL), electron microscopy on postoperative day 1 revealed retinal tissue edema, including intracellular edema and extracellular edema. While this edema gradually subsided, residual RNFL edema persisted at 4 weeks post-injection [10]. Notably, these toxic effects were not universally observed. In contrast, Young et al. reported no detectable toxicity in rabbit eyes receiving intravitreal GCV 1 mg/0.1mL, with preserved retinal structure and function confirmed through funduscopic examinations, electroretinography, and histologic analyses [19]. 

Pharmacokinetic studies of intravitreal GCV revealed retinal GCV concentrations surpassing vitreous levels by 24 h [20], with RPE cells identified as the primary clearance pathway [21]. Both systemic and intravitreal administration demonstrated drug accumulation in photoreceptor outer segments and RPE cells [22], further confirming RPE’s central role in ocular GCV metabolism.

The animal models discussed above resemble clinical scenarios where CMVR is treated with intravitreal GCV monotherapy. However, in real-world practice, GCV treatment strategies for CMVR vary and include intravitreal injection monotherapy, systemic administration monotherapy, combined systemic and intravitreal administration, or intravitreal therapy following systemic treatment discontinuation. Dosages for intravitreal injections range widely (2–6 mg/0.05–0.1 mL, weekly or twice a week) [23, 24], Notably, aside from isolated case reports, most clinical studies have not reported GCV-related complications or toxicity in these regimens. According to the 2024 Guidelines for Opportunistic Infections in AIDS, combining systemic therapy with intravitreal GCV is recommended based on pharmacokinetic considerations. However, the clinical benefits of this adjunctive approach remain unproven in clinical trials. While intravitreal injections deliver immediate high drug concentrations to the lesion, systemic administration achieves steady-state intraocular drug levels over time [25]. This dual administration may lead to overlapping concentration gradients, altered tissue penetration dynamics, and unpredictable drug accumulation or elimination rates in retinal tissues.

In our study, the investigated cases involved concurrent intravenous and intravitreal GCV administration. As per the prescribing information, the concentration of GCV for intravenous infusion must not exceed 10 mg/mL. The intravitreal concentration (2.5 mg/0.05 mL equals 50 mg/mL) used in our cases represents an ultra-high-concentration formulation. Notably, the strongly alkaline solution with a pH of 10–11 aligns with prior reports [26]. We hypothesize that the high-concentration alkaline GCV solution may induce acute chemical irritation to RPE, and cumulative GCV exposure from repeated intravitreal injections and combined systemic therapy could lead to chronic damage to RPE cells.

#### Systemic and ocular contributors

The reversible blood-retinal barrier (BRB) disruption (e.g., acute SRF and hyperreflective material) aligns with known HIV-driven vascular pathology. Firstly, the envelope glycoprotein gp120, exposed on the surface of the HIV envelope, is essential for virus entry into cells by the attachment to specific cell surface receptors. The BRB disruption by glycoprotein gp120 has been widely recognized. These mechanisms include downregulating tight junction proteins (ZO-1, occludin) in RPR cells [27, 28]. Secondly, AIDS-accompanying anemia and hypoalbuminemia could exacerbate microvascular changes [29, 30]. Thirdly, nucleotide reverse transcriptase inhibitors (NRTIs), which are used to inhibit polymerase and treat HIV, can lead to decreased mitochondrial DNA in RPE cells, appearing as RPE mottling and atrophy [31]. 

Another vital interpretation could be the ocular inflammatory milieu. Pro-inflammatory cytokines (IL-1α, IL-1β, TNF-α) that induce inflammatory damages are elevated in eyes with CMVR [32, 33]. These inflammatory damages will gradually worsen as the immune reconstitution after the initiation of ART, which is called immune reconstitution inflammatory syndrome (IRIS). IRIS is a complex interaction between immune system restoration and tissue-destructive inflammatory responses. Clinically, ocular IRIS can be transient and self-limiting and present with cystoid macular edema. For patients with AIDS-related CMVR, IRIS can manifest as immune recovery uveitis (IRU) with inactive CMVR and CMV-IRR with active CMVR [34, 35]. Notably, the patient’s immune reconstitution can paradoxically amplify inflammatory responses to aggravate the RPE damage [36]. In this study, three out of five patients (five out of seven eyes) belong to CMV-IRR. OCT images demonstrate ONL edema, subretinal fluid, and RPE atrophy. These changes could be due to aggravating inflammatory responses after injection stimulation.

In this compromised state, even therapeutic GCV injections, with further transient osmolality or pH fluctuations, may tip the balance toward clinically apparent edema.

### Implications for next-generation therapies

This framework emphasizes balancing therapeutic efficacy with safety, advocating for tailored strategies based on disease severity, anatomical involvement, and patient-specific risks.

Implications for next-generation therapies may include: for macula-sparing CMVR cases, consider systemic anti-CMV monotherapy first; for patients with macular-sparing CMVR and cannot tolerate systemic anti-CMV therapy, close postoperative monitoring (via fundus examination and OCT) is critical within the first 24 h post-injection; in severe CMVR cases requiring combined systemic and intravitreal GCV therapy, intravitreal injections should be discontinued once retinal lesions stabilize to minimize cumulative toxicity; Novel intravitreal GCV formulations should account for local chemical irritation and the risk of chronic retinal damage from drug accumulation. Preclinical studies must evaluate both acute and long-term retinal toxicity.

The small sample size of cases limits the current study, and future studies should aim to identify more cases of this condition. The current study is a retrospective observation, and comprehensive fundus examinations were limited, such as the visually evoked potential, electroretinography, fundus fluorescein angiography, or coherence tomography angiography. Furthermore, the retrospective analysis of clinical cases lacked strict OCT assessment in the immediate post-injection phases and follow-ups. Consequently, some transient acute macular edema may be missed, and the actual rate could be higher than reported. Future prospective and controlled studies are needed to investigate this phenomenon and to clarify the true causality.

## Conclusion

This study highlights a novel insight into the safety of intravitreal GCV delivery. GCV-related complications could manifest as acute macular edema and chronic RPE degeneration. These changes suggest specific RPE stress from complex interactions of drug effects, HIV-driven barrier disruption, and ocular inflammatory destruction rather than a direct effect of GCV alone. This study underscores the imperative to explore safer GCV strategies for CMVR while emphasizing the integration of comprehensive safety profiles into the development of novel antiviral agents.

## Supplementary Information

Supplementary Material 1. Supplemental Figure 1: CMVR without macular involvement in the right eye of Case 1; OCT image before the incident injection; On the first day after the 6th intravitreal ganciclovir injection, the patient experienced impaired vision, with OCT revealing a retinal nerve fiber layer fold and macular subretinal fluid; Two weeks later, macular subretinal fluid resolved with VA recovery, but a residual retinal nerve fiber layer fold persisted

Supplementary Material 2. Supplemental Figure 2: CMVR without macular involvement in the left eye of Case 2. OCT image before the incident injection. Two hours after the first intravitreal ganciclovir injection, the patient experienced impaired vision, with OCT revealing outer nuclear layeredema and macular subretinal fluid. On postoperative day 3, the ONL edema resolved, but macular subretinal fluid persisted. At 5 weeks post-injection, macular subretinal fluid resolved, and OCT showed normal macular structure

Supplementary Material 3. Supplemental Figure 3: CMVR without macular involvement in the right eye of Case 3. OCT image before the incident injection. On the first day after the 3rd intravitreal ganciclovir injection, the patient reported impaired vision, with OCT demonstrating outer nuclear layeredema, macular subretinal fluid, and hyperreflective foci. On postoperative day 3, the outer nuclear layeredema resolved, but macular subretinal fluid and hyperreflective foci remained. At 1 week post-injection, ONL edema and subretinal fluid resolved, with residual hyperreflective foci. The right eye received anti-vascular endothelial growth factor therapy; OCT at 5 weeks post-treatment showed restored macular architecture

Supplementary Material 4. Supplemental Figure 4: CMVR without macular involvement in the left eye of Case 3. OCT image before the incident injection. On the first day after the 3rd intravitreal ganciclovir injection, the patient experienced impaired vision, with OCT revealing macular subretinal fluid. Subsequent intravitreal injections were discontinued. At 1 week post-injection, OCT showed resolved subretinal fluid and restored macular architecture

Supplementary Material 5. Supplemental Figure 5:CMVR without macular involvement in the left eye of Case 4. OCT image before the incident injection. On the first day after the 5th intravitreal ganciclovir injection, the patient reported severe vision loss, with OCT showing macular subretinal fluid and vertical hyperreflective foci. Subsequent injections were discontinued. At 3 weeks post-injection, OCT demonstrated restored macular architecture

Supplementary Material 6. Supplemental Figure 6:The pH testing of the intravitreal ganciclovir solutionwas used in this study. A mixture of 5 mL normal saline and 250 mg ganciclovir powder was tested with pH test strips from two different manufacturers, both showing a pH of 10 - 11

## Data Availability

Data that support the findings of this study are available from the corresponding author upon reasonable request and approval.
