# Supplementary figures and images for: Retinal pigment epithelium stress following intravitreal ganciclovir: a novel insight from clinical spectrum
Source: J Ophthalmic Inflamm Infect. 2025 Sep 26;15:71. doi: 10.1186/s12348-025-00532-3 (PMC12474833; doi:10.1186/s12348-025-00532-3)

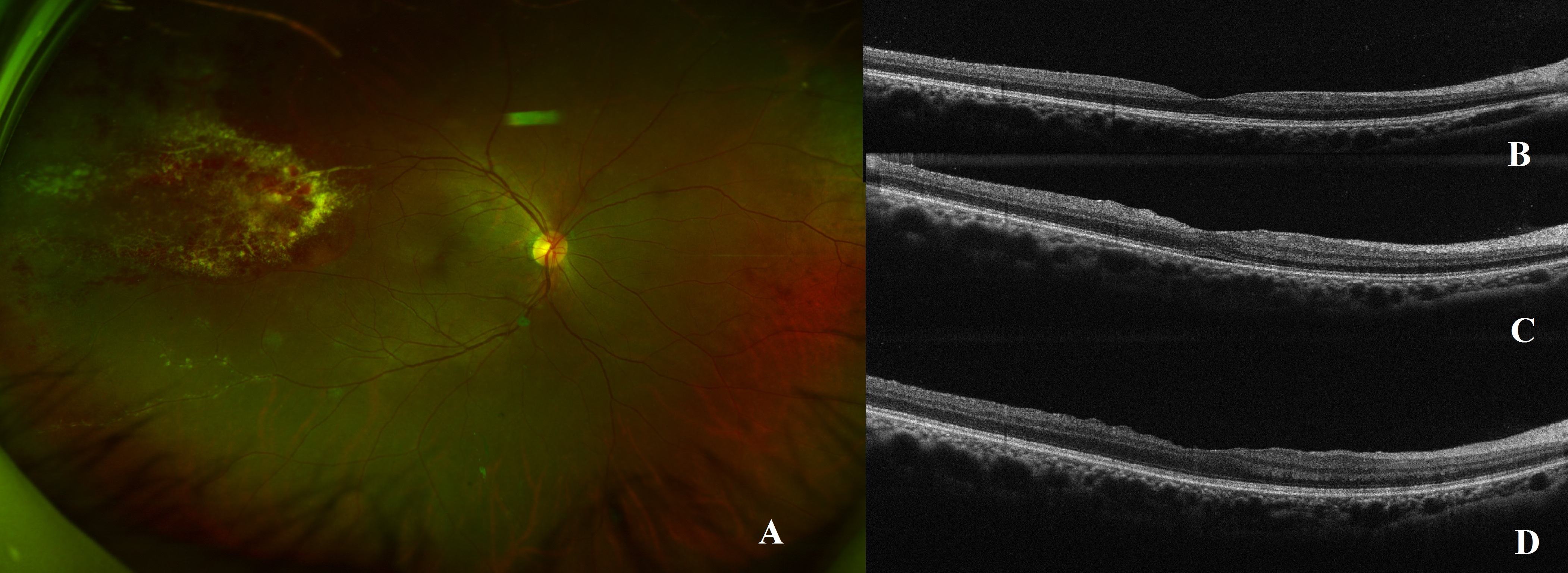

Supplement: Supplementary file 1 — Supplementary Material 1. Supplemental Figure 1: CMVR without macular involvement in the right eye of Case 1; OCT image before the incident injection; On the first day after the 6th intravitreal ganciclovir injection, the patient experienced impaired vision, with OCT revealing a retinal nerve fiber layer fold and macular subretinal fluid; Two weeks later, macular subretinal fluid resolved with VA recovery, but a residual retinal nerve fiber layer fold persisted [file 12348_2025_532_MOESM1_ESM.jpg]

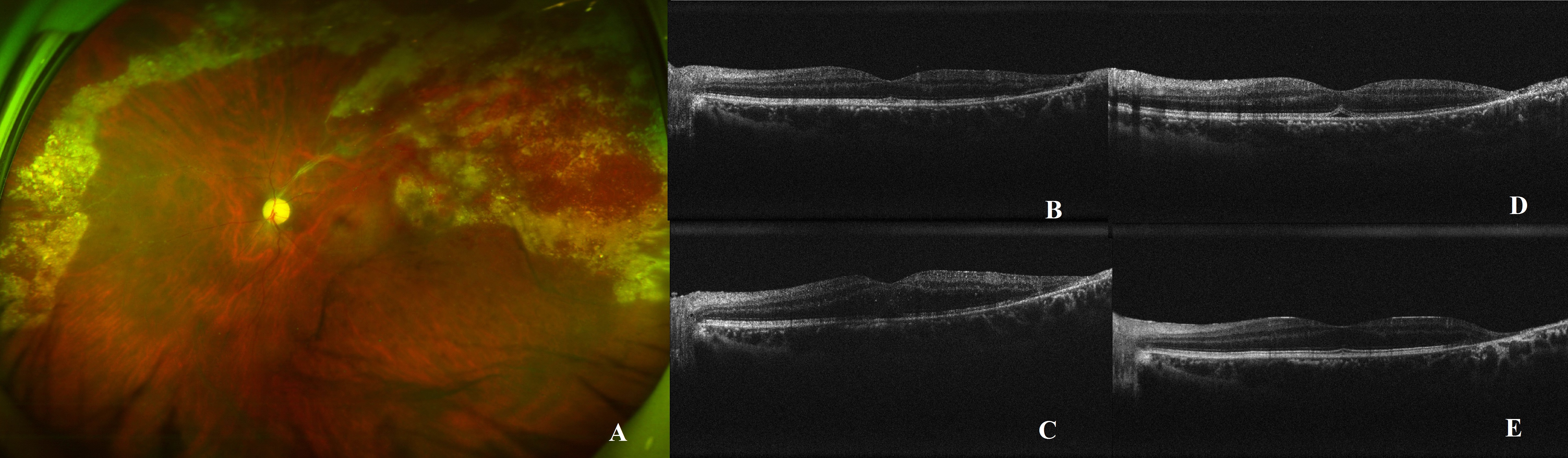

Supplement: Supplementary file 2 — Supplementary Material 2. Supplemental Figure 2: CMVR without macular involvement in the left eye of Case 2. OCT image before the incident injection. Two hours after the first intravitreal ganciclovir injection, the patient experienced impaired vision, with OCT revealing outer nuclear layeredema and macular subretinal fluid. On postoperative day 3, the ONL edema resolved, but macular subretinal fluid persisted. At 5 weeks post-injection, macular subretinal fluid resolved, and OCT showed normal macular structure [file 12348_2025_532_MOESM2_ESM.jpg]

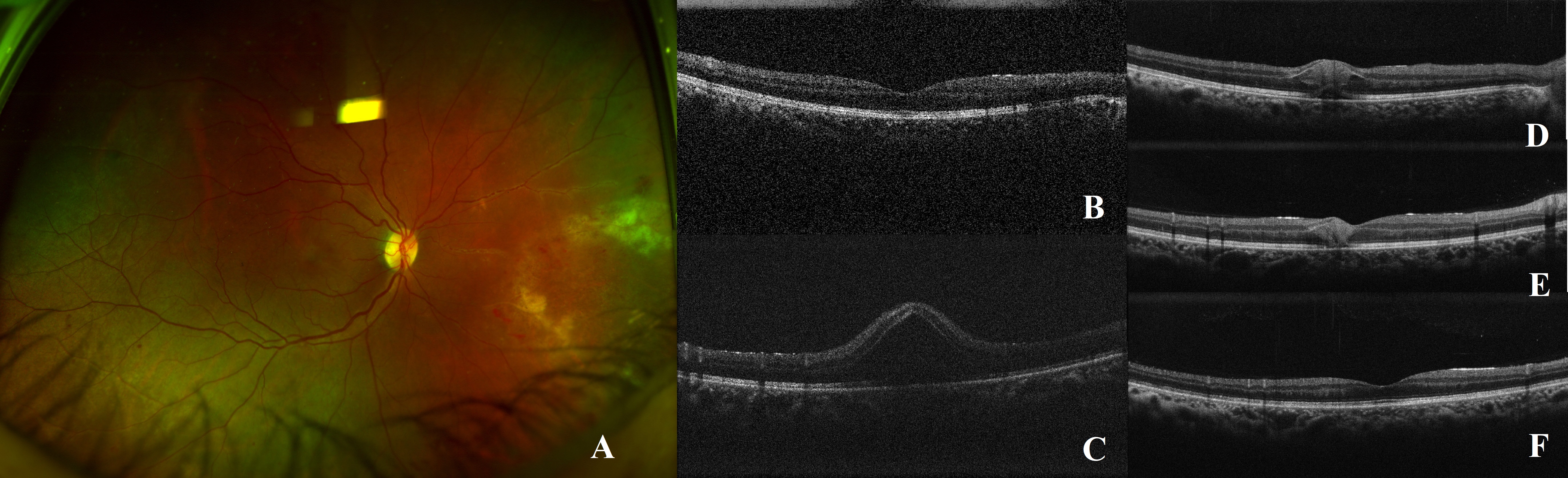

Supplement: Supplementary file 3 — Supplementary Material 3. Supplemental Figure 3: CMVR without macular involvement in the right eye of Case 3. OCT image before the incident injection. On the first day after the 3rd intravitreal ganciclovir injection, the patient reported impaired vision, with OCT demonstrating outer nuclear layeredema, macular subretinal fluid, and hyperreflective foci. On postoperative day 3, the outer nuclear layeredema resolved, but macular subretinal fluid and hyperreflective foci remained. At 1 week post-injection, ONL edema and subretinal fluid resolved, with residual hyperreflective foci. The right eye received anti-vascular endothelial growth factor therapy; OCT at 5 weeks post-treatment showed restored macular architecture [file 12348_2025_532_MOESM3_ESM.jpg]

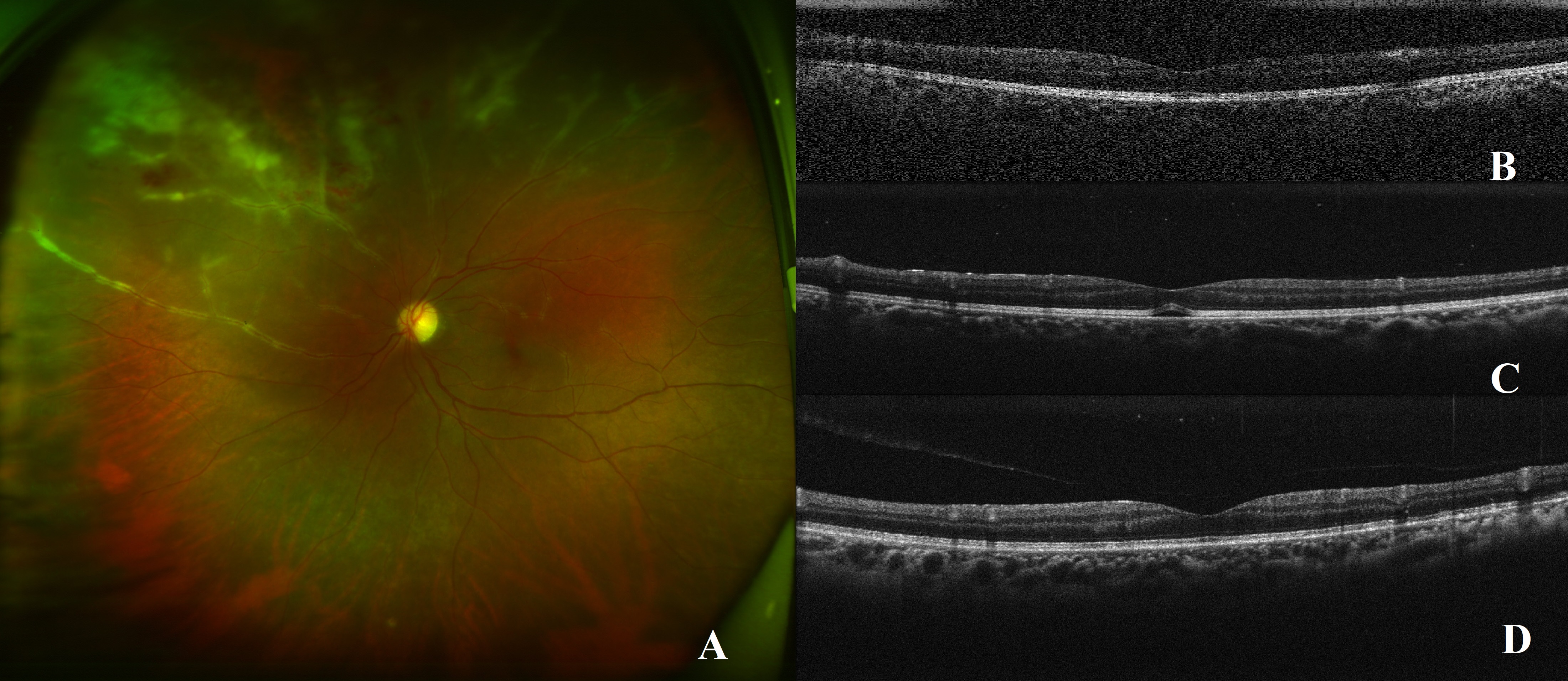

Supplement: Supplementary file 4 — Supplementary Material 4. Supplemental Figure 4: CMVR without macular involvement in the left eye of Case 3. OCT image before the incident injection. On the first day after the 3rd intravitreal ganciclovir injection, the patient experienced impaired vision, with OCT revealing macular subretinal fluid. Subsequent intravitreal injections were discontinued. At 1 week post-injection, OCT showed resolved subretinal fluid and restored macular architecture [file 12348_2025_532_MOESM4_ESM.jpg]

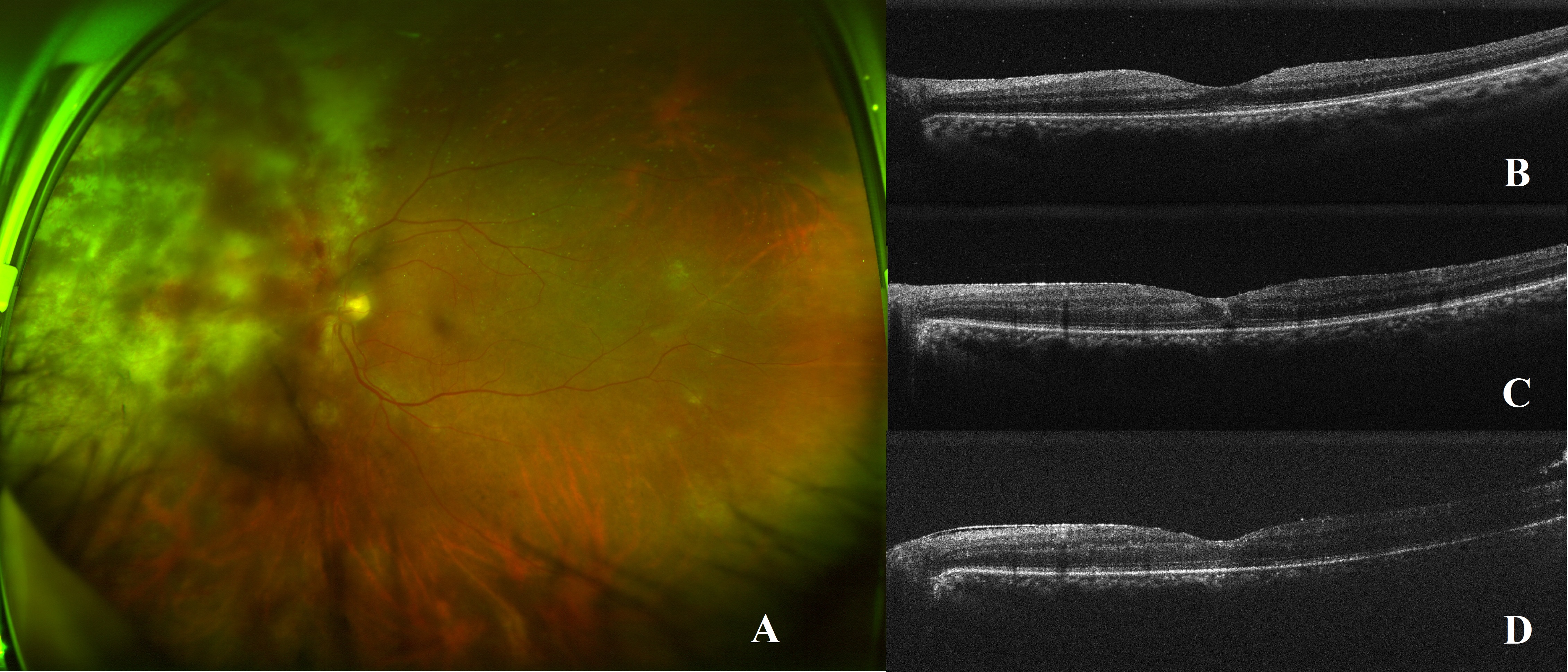

Supplement: Supplementary file 5 — Supplementary Material 5. Supplemental Figure 5:CMVR without macular involvement in the left eye of Case 4. OCT image before the incident injection. On the first day after the 5th intravitreal ganciclovir injection, the patient reported severe vision loss, with OCT showing macular subretinal fluid and vertical hyperreflective foci. Subsequent injections were discontinued. At 3 weeks post-injection, OCT demonstrated restored macular architecture [file 12348_2025_532_MOESM5_ESM.jpg]

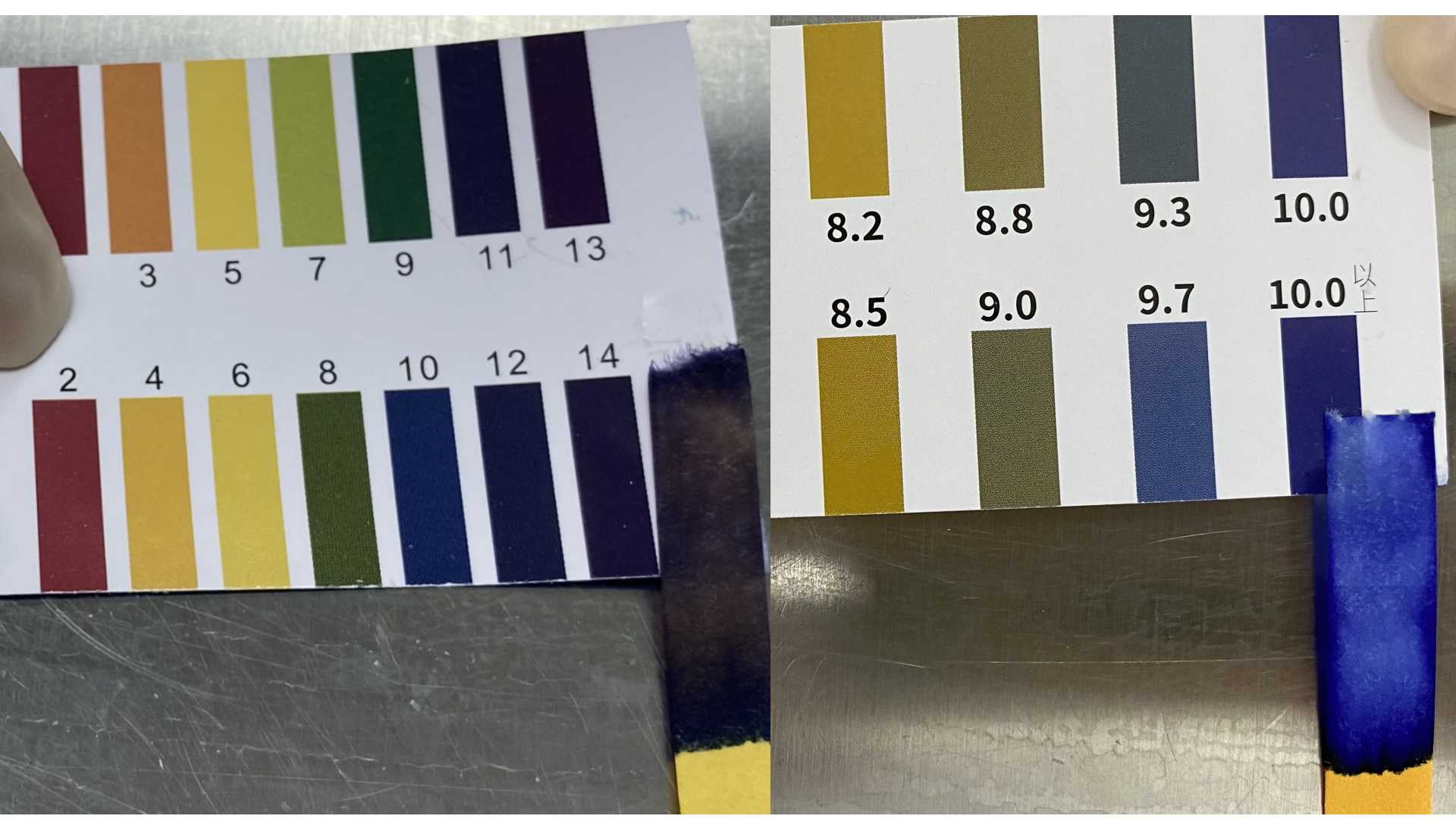

Supplement: Supplementary file 6 — Supplementary Material 6. Supplemental Figure 6:The pH testing of the intravitreal ganciclovir solutionwas used in this study. A mixture of 5 mL normal saline and 250 mg ganciclovir powder was tested with pH test strips from two different manufacturers, both showing a pH of 10 - 11 [file 12348_2025_532_MOESM6_ESM.jpg]
